# Klotho revisited: tubular heterogeneity reshapes mineral metabolism, aging, and CKD

**DOI:** 10.1007/s00424-026-03197-6

**Published:** 2026-07-20

**Authors:** Ganesh Pathare

**Affiliations:** Bone-Kidney Axis and Regeneration Laboratory, Dept. of Infectious Diseases and Public Health, Jockey Club College of Veterinary Medicine and Life Sciences, City University of Hong Kong, To Yuen Street 31, Kowloon Tong, 999077 Hong Kong SAR China

**Keywords:** Klotho, FGF23, Proximal tubule, Distal convolution, Mineral metabolism, Chronic kidney disease

## Abstract

α-Klotho (hereafter Klotho) was discovered as an aging-suppressor protein whose function is intrinsically dependent on the kidney. Its shed ectodomain, soluble Klotho (sKlotho), is detectable in blood and urine. As an obligate co-receptor for endocrine fibroblast growth factor 23, Klotho operates at the intersection of mineral metabolism and chronic kidney disease (CKD). Within the kidney, Klotho expression is moderate in the proximal tubule but strongly enriched in the distal nephron, although the functional significance of this heterogeneity remains unclear. Recent findings from nephron segment-specific Klotho knockout mice reveal functional specialization of tubular Klotho along the nephron. According to the revised model, distal nephron Klotho regulates calcium reabsorption, bone remodeling, and urinary sKlotho levels. By contrast, proximal tubular Klotho regulates phosphate and vitamin D metabolism and is likely the principal source of circulating sKlotho; its loss recapitulates hyperphosphatemia, FGF23 resistance, and ageing-like phenotypes. This review re-evaluates the long-standing paradigm of renal Klotho biology in light of emerging evidence for functionally distinct proximal and distal nephron Klotho. Together, these insights place the kidney and its tubular Klotho heterogeneity at the center of mineral metabolism, aging and CKD.

## Introduction

The nephron, the functional unit of the kidney, is organised into functionally distinct tubular segments that are lined by specialised epithelial cells. The proximal nephron is the principal site of solute reabsorption, reclaiming the majority of filtered ions, glucose, amino acids, and bicarbonate. Phosphate (Pi), a key mineral for skeletal mineralization, cellular metabolism, and intracellular signaling, is also reabsorbed predominantly in the proximal tubule, but its handling is tightly hormonally regulated rather than reflecting constitutive bulk uptake. The distal nephron performs fine-tuned regulation of Ca^2+^, Na^+^, and K^+^. The discovery of α-Klotho (hereafter Klotho) in 1997 added a striking new dimension as it was found to be expressed predominantly in the distal nephron, an unexpected restriction for a protein whose loss produced a syndrome of accelerated systemic aging [1]. It took over a decade to establish that Klotho is also expressed in the proximal nephron, albeit at lower levels [2]. The functional significance of this spatial gradient of Klotho expression has long puzzled nephrologists and mineral metabolism researchers [1–6].

Klotho is predominantly expressed in the kidney, with substantial expression in the parathyroid glands and choroid plexus, and lower levels in a few other tissues [1]. Critically, kidney-specific Klotho deletion recapitulates the full premature aging phenotype of global knockout mice, establishing the kidney as the indispensable site of Klotho action [4]. Klotho is a transmembrane protein with a large extracellular domain. Its soluble form (sKlotho), generated by proteolytic ectodomain shedding, is detectable in blood, urine, and cerebrospinal fluid [6]. An alternatively spliced transcript with a premature stop codon is not translated, making proteolytic shedding the primary source of sKlotho [7]. A pivotal breakthrough in understanding Klotho function came in 2006, when it was identified as the obligate co-receptor for the bone-derived phosphaturic hormone fibroblast growth factor 23 (FGF23) [8]. Current structural studies indicate the formation of an asymmetric FGF23–FGF receptor–Klotho–heparan sulfate (HS) quaternary complex with a 1:2:1:1 stoichiometry, which activates MAPK/ERK signalling [9, 10]. Beyond its co-receptor function, sKlotho has been proposed to exert FGF23-independent endocrine and paracrine actions [2, 6].

### Tubular heterogeneity of Klotho

**Fig. 1:**
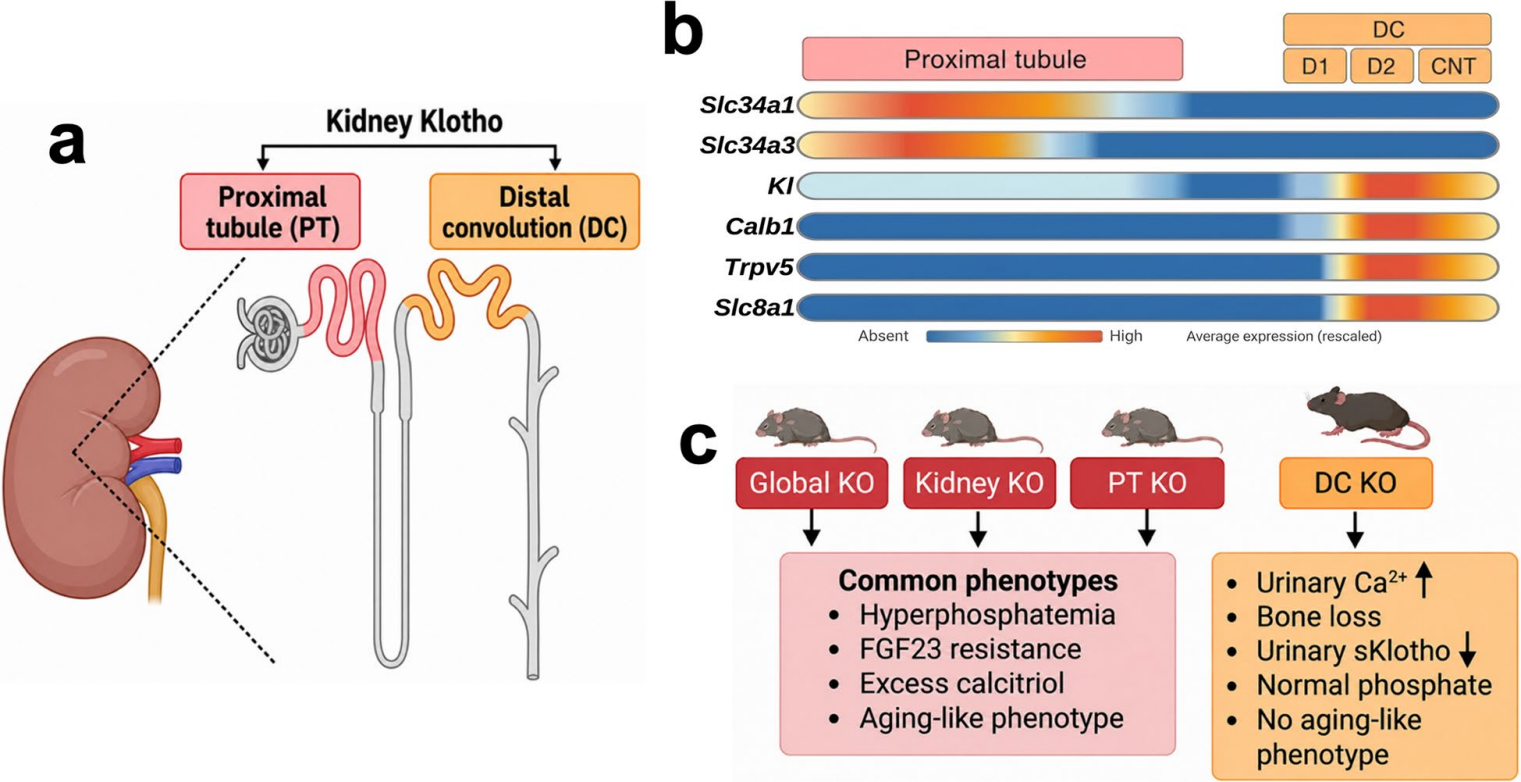
Klotho exhibits nephron segment-specific roles in mineral metabolism. (**a**) Schematic of the mouse nephron illustrating Klotho in proximal tubule (red) and in distal convolution (orange), comprising DCT1 (D1), DCT2 (D2), and connecting tubule (CNT). (**b**) scRNA-seq expression analysis from *Kidney Cell Explorer* (https://cello.shinyapps.io/kidneycellexplorer/) showing co-enrichment of *Kl* with Ca^2+^ transport genes (*Trpv5, Calb1, Slc8a1*) in DCT2/CNT, contrasting with PT-enriched *Slc34a1/Slc34a3*. (**c)** Phenotypic consequences of nephron segment–specific Klotho deletion. Global, kidney, and PT knockout mice share hyperphosphatemia, FGF23 resistance, and an aging-like phenotype. DC-specific knockout produces hypercalciuria, reduced bone mineral density, and loss of urinary sKlotho without Pi dysregulation

As discussed earlier, within the kidney, Klotho expression is modest in the renal proximal tubule (PT) but high in the distal convolution (DC) that includes the distal convoluted tubule (DCT) and the connecting tubule (CNT) (Fig. 1a/b). The DCT is divided into an early portion (DCT1) and a late portion (DCT2), which ends in the CNT. Using scRNA-seq on isolated murine DC cells, it was recently demonstrated that *Kl* transcripts are more abundant in DCT2/CNT than in DCT1 [5]. Immunofluorescence of kidneys with nephron segment-specific markers confirmed this pattern at the protein level. Overall, these findings highlight a marked tubular heterogeneity of Klotho expression along the nephron, with modest expression in the PT but higher abundance along the DC segment, reaching its highest levels in the DCT2/CNT region (Fig. 1b). This spatial enrichment suggests that Klotho-dependent signaling and transport processes are probably differentially regulated across nephron segments.

### DC Klotho as the Ca^2+^ regulatory module

The intra-DC gradient of Klotho is biologically meaningful. DCT2 cells resemble CNT cells and differ from DCT1 cells by exhibiting higher expression levels of proteins involved in transcellular Ca^2+^ transport — TRPV5, calbindin-D28k, NCX1, VDR, PTH1R, and KLK1, all of which participate in the hormonal regulation of Ca^2^⁺ handling [5]. The enrichment of Klotho precisely in the segment that performs the transcellular Ca^2+^ reabsorption is not coincidental (Fig. 1b). In mice lacking Klotho throughout the DC, RNA-seq of sorted DC tubules revealed coordinated downregulation of *Trpv5*, *Calb1*, *Vdr*, *Pth1r*, and *Klk1*, alongside suppressed MAPK signalling [5]. Mouse models lacking Klotho in both DCT2/CNT and entire DC exhibited a similar degree of hypercalciuria and TRPV5 loss, confirming that the Ca^2+^ phenotype is primarily linked to Klotho in DCT2/CNT segment. Consequently, the resulting hypercalciuria translated into skeletal abnormalities, including reduced bone mineral density (Fig. 1c). RNA-seq of Klotho-deficient DC, compared with a recent transcriptomic analysis of the temporal ERK dynamics of in vitro FGF23–FGFR–Klotho signalling [11], revealed selective MAPK pathway engagement. Late ERK target genes were significantly downregulated, whereas early ERK target genes remained unaffected. Because early ERK targets are induced primarily by acute FGF23 exposure, while late ERK targets reflect sustained FGFR–Klotho signalling, these findings indicate that the ERK transcriptomic changes in Klotho-deficient DC result from the loss of tonic FGF23–FGFR–Klotho signalling. Overall, the most parsimonious interpretation is that tonic FGF23-FGFR-Klotho signalling in DCT2/CNT normally sustains MAPK activity, which in turn maintains the transcriptional programme governing Ca^2+^ transport, and loss of Klotho collapses the downstream gene regulatory network. The reasons for the modest expression of Klotho in the DCT1 are unclear, but it may participate in Na^+^ reabsorption via the sodium-chloride cotransporter and regulate arterial blood pressure [5].

The FGF23–Klotho complex was previously also shown to regulate TRPV5 at the post-translational level via ERK1/2, SGK1, and WNK4 [12]. On the other hand, earlier studies proposed that urinary sKlotho stabilizes and stimulates TRPV5 activity from the apical side through its enzymatic activity [6]. However, structural studies suggest that sKlotho may lack intrinsic enzymatic activity [10]. Definitive in vivo evidence for the effect of sKlotho enzymatic activity on TRPV5 is still lacking.

### PT Klotho regulates Pi homeostasis

Targeted and precise deletion of Klotho in the DC does not alter serum or urinary Pi levels or induce FGF23 resistance in mice [5]. In contrast, a previous study using Cdh16-Cre–mediated deletion of Klotho reported hyperphosphatemia and FGF23 resistance [3]; probably because Cdh16-Cre drives recombination broadly throughout the nephron, including the PT. A second study using PT-targeted deletion with three inducible Cre lines yielded incomplete recombination with mild or no hyperphosphatemia under basal conditions, with Pi dysregulation emerging only upon dietary Pi loading [13]. These discrepancies among mouse models examining the role of DC/PT Klotho in Pi homeostasis likely reflect differences in Cre-driver specificity, recombination efficiency, and the floxed Klotho mouse strains used. The rationale for implicating DC Klotho in phosphate regulation stemmed from the long-held purported hypothesis that the DC secretes sKlotho as a paracrine factor acting on the PT [3, 6, 13]. However, recent data suggest that DC-derived sKlotho is primarily secreted into the urine [5].

The PT is the principal nephron segment responsible for transcellular Pi reabsorption via apical NaPi-IIa/IIc transporters and for the formation of active vitamin D (calcitriol) through Cyp27b1. It is likely the primary site where FGF23–Klotho signaling suppresses Pi reabsorption and reduces calcitriol synthesis. The convergence of anatomical expression, transport physiology, and genetic loss-of-function data therefore places PT Klotho, not DC-derived Klotho, as the obligate co-receptor for FGF23 endocrine action on Pi and vitamin D metabolism. This is supported by another in vivo study, as PT-specific deletion of Klotho led to hyperphosphatemia, FGF23 resistance, excess calcitriol levels and induced ectopic calcification and premature aging phenotype [14]. Notably, these phenotypes closely resemble those observed in kidney-specific or global Klotho knockout mice [1, 4, 5, 14]. Thus, Klotho deletion that includes the PT likely causes hyperphosphatemia, FGF23 resistance and aging-like phenotype (Fig. 1c). Overall, current evidence favors PT Klotho as the physiologically relevant regulator of FGF23-dependent Pi homeostasis.

### Dichotomy of sKlotho secretion by DC and PT

One important emerging concept is that DC-derived Klotho appears to be secreted predominantly into the urine rather than the systemic circulation [5]. The observation that DC-specific Klotho deletion does not alter circulating sKlotho levels, despite an almost complete reduction in urinary sKlotho, was unexpected but unequivocal [5]. On the other hand, inducible kidney-specific Klotho deletion resulted in the loss of both circulating and urinary sKlotho [5]. Constitutive kidney-specific Klotho deletion in an earlier study also resulted in an approximately 80% reduction in serum sKlotho levels, but urinary sKlotho was not measured [4]. The combined findings support the model that the PT may represent a major source of circulating sKlotho, whereas the DC secretes Klotho into the urine [5]. However, definitive experimental validation is still lacking. Polarised primary cultures or tubuloids could in principle resolve directionality of sKlotho secretion from PT and DC, but no robust system currently maintains endogenous Klotho expression in PT or DC cells in stable polarised monolayers. This dissociation raises a key question: what is the physiological function of sKlotho in blood and urine, which is explored further in the open questions below.

Early immunoelectron microscopy detected Klotho at the basolateral membrane and cytoplasm of DC cells, and at both apical and basolateral membranes of PT cells [2]. More recent immunofluorescence data suggest that Klotho expression is observed throughout the cytoplasm and it is slightly higher at the apical membrane in DC cells, whereas in PT cells it is distributed throughout the cytoplasm [5]. Higher-resolution in vivo localization studies are needed to clarify the precise subcellular localization of Klotho, which may help in understanding segment-specific sKlotho secretion.

### Open questions and future directions

#### i) PT and DC Klotho: divergent abundance, FGF receptors, and downstream effectors

Why per-cell Klotho abundance is low in the PT but high in the DC remains unclear [2, 5]. This tubular heterogeneity likely reflects functional specialisation of the nephron that emerged during vertebrate evolution. One possibility is that transcellular Ca^2+^ transport in DCT2/CNT demands higher per-cell Klotho density to sustain tonic MAPK output across a broad downstream transcriptional programme, whereas phosphaturic signalling in the PT may be efficiently driven at lower Klotho levels [5].

**Fig. 2:**
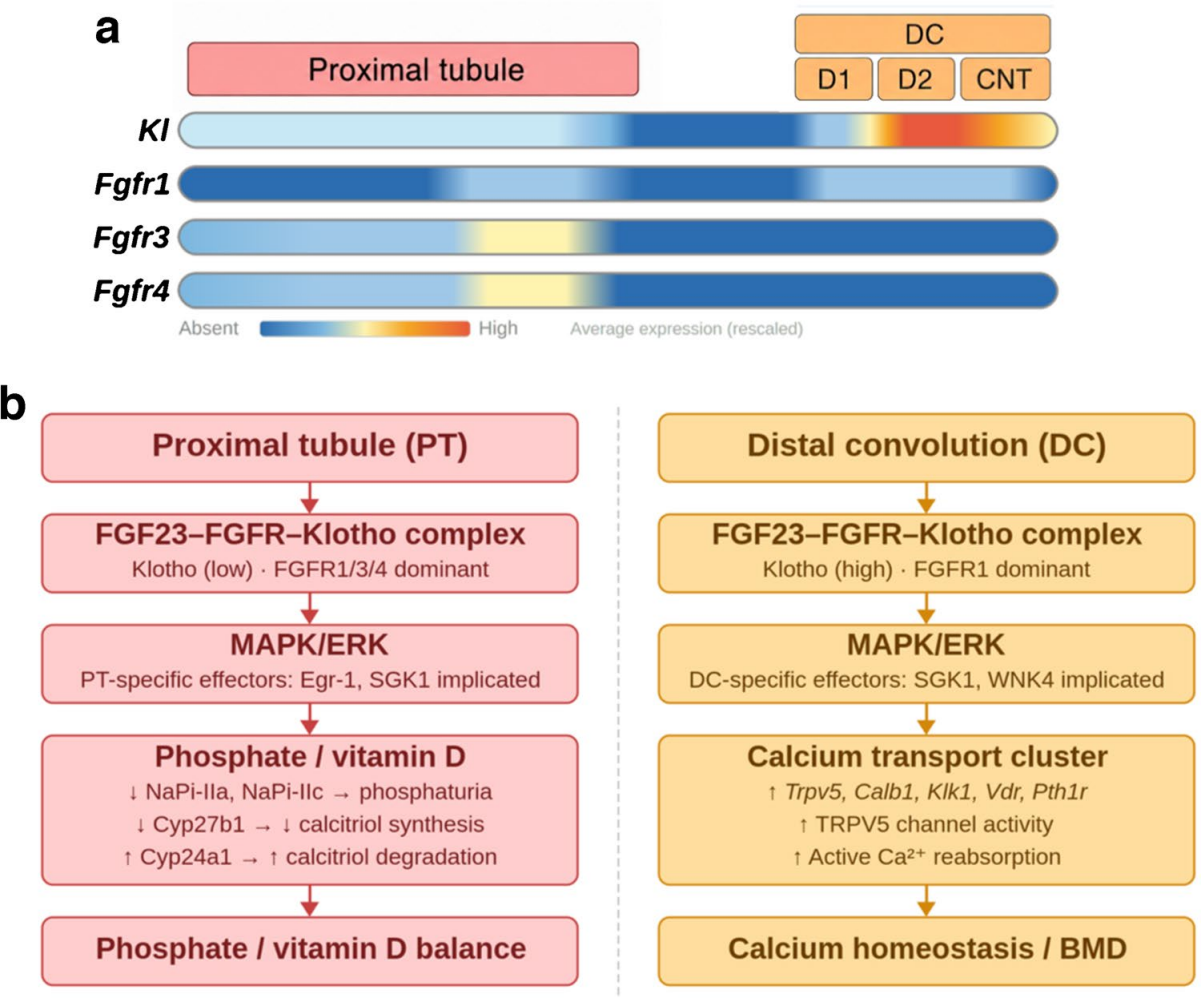
FGF23–Klotho signalling and potential role of differential FGFRs in the proximal tubule and distal convolution. (**a**) Rescaled average expression of *Kl, Fgfr1, Fgfr3*, and *Fgfr4* along the nephron based on scRNA-seq of kidney. *Fgfr3* and *Fgfr4* predominate in the PT, with moderate *Fgfr1* in the second half of the PT. The DC mainly expresses *Fgfr1*. (**b**) In the PT (red), orchestration of FGFR1/3/4 probably favours low-Klotho-mediated MAPK/ERK activation and suppresses NaPi-IIa/IIc and inhibits *Cyp27b1* while inducing *Cyp24a1*, reducing calcitriol synthesis. In the DC (orange), high Klotho drives FGFR1-dominant MAPK/ERK signalling and upregulates transcripts for the Ca^2+^ transport cluster (*Trpv5, Calb1, Klk1, Vdr, Pth1r*), TRPV5 channel activity, and therefore active Ca^2^⁺ reabsorption, regulating bone mineral density (BMD). The calcitriol regulation in PT indeed indirectly regulates Ca^2+^ reabsorption and BMD as well.

The PT co-expresses FGFR1/3/4, while the DC is dominated exclusively by FGFR1 (Fig. 2a). FGFR2 is excluded because structural studies do not support its engagement by Klotho, and no in vivo studies have implicated it in renal Klotho-dependent signalling [9, 10]. In vivo, FGFR1 is the dominant receptor mediating FGF23-induced phosphaturia [16], with FGFR4 also contributing [17]. FGFR3 and FGFR4 jointly mediate FGF23-dependent suppression of calcitriol synthesis in the PT [17, 18]. It is conceivable that the multiplicity of FGFRs in the PT allows FGF23 signalling to proceed efficiently at low Klotho abundance. By contrast, exclusive reliance on FGFR1 in the DC may require higher Klotho abundance to achieve comparable receptor complex formation and downstream signalling output. This may lead to differences in receptor affinity, complex stoichiometry, or downstream adaptor coupling (Fig. 2b). These remain speculative, and direct experimental evidence for differential FGFR contributions to PT versus DC Klotho signalling is lacking.

The downstream effectors linking MAPK activation to NaPi-IIa/IIc suppression in the PT may include EGR1 and SGK1, whereas SGK1 and WNK4 have been implicated in the DC [12, 15]. How these mediators precisely and differentially regulate segment-specific mineral metabolism remains incompletely understood [2, 5, 12, 15].

#### ii) Membrane-bound Klotho versus sKlotho: unresolved physiology

The classical model of FGF23 signalling positions membrane-bound Klotho as the obligate co-receptor within a cell-surface complex on tubular epithelial cells [8]. However, sKlotho has also been proposed to serve as a soluble co-receptor, enabling FGF23 signalling [10]. Structural studies have shown that sKlotho can substitute for membrane-bound Klotho to stabilize the FGF23–FGFR complex in a 1:2:1:1 stoichiometry [9, 10]. However, the in vivo relevance of sKlotho as a co-receptor remains an open and actively debated question [5, 6, 10]. Circulating sKlotho in humans and rodents has been estimated to be in the low picomolar range, orders of magnitude below the concentrations used in most cell-based sKlotho rescue experiments [2, 10]. Whether such low circulating levels are sufficient to assemble a functional FGF23–FGFR–sKlotho complex in target tissues remains unresolved [6]. It has nonetheless been reported that in CKD models, where membrane-bound Klotho is markedly depleted, sKlotho treatment reverses disease progression [6, 19].

Segment-specific knockout data are informative regarding the relative contribution of membrane-bound versus sKlotho in regulating Ca^2^⁺ homeostasis. DC-specific Klotho deletion impairsthe Ca^2+^ transport transcriptional programme in DCT2/CNT despite preserved circulating sKlotho levels [5]. Moreover, mice lacking Klotho selectively in DCT2/CNT exhibit a similar degree of TRPV5 loss and hypercalciuria as mice lacking Klotho throughout the entire DC, despite the latter having virtually no urinary sKlotho while DCT2/CNT-specific knockouts retain partially preserved urinary sKlotho [5]. This dissociation between sKlotho levels and Ca^2+^ phenotype severity argues against the direct effect of sKlotho and further supports membrane-bound Klotho as the functionally relevant form in DCT2/CNT. Further work is needed to dissect roles of membrane-bound and sKlotho.

#### iii) Consequences of differential urinary and serum sKlotho pools

Although the urinary sKlotho concentration substantially exceeds that of serum, the functional significance of urinary sKlotho remains largely unknown [5]. DC-specific knockout mice lack virtually all urinary sKlotho and show no overt metabolic phenotype under standard housing conditions. DC-specific Klotho knockout mice also exhibit severe hypercalciuria, a major risk factor for Ca^2+^ nephrolithiasis [5]. Whether combined sKlotho deficiency and hypercalciuria predispose to nephrolithiasis remains unknown and represents an important knowledge gap.

The potential effects of circulating sKlotho have been extensively studied and the ongoing debate regarding the distinct functions of membrane-bound Klotho versus sKlotho is discussed earlier. If the PT is indeed the major source of circulating sKlotho, this reframes its role as a CKD biomarker. Circulating sKlotho declines in CKD, whereas urinary sKlotho has been far less studied [6]. If mouse data hold true in humans, circulating sKlotho may primarily reflect PT Klotho loss, whereas urinary sKlotho may reflect DC Klotho loss [5]. Since PT injury drives hyperphosphatemia, which in turn aggravates CKD progression, circulating sKlotho may have greater predictive value for disease progression. This PT/DC dichotomy suggests that serum and urinary sKlotho should be interpreted as distinct biomarkers rather than interchangeable measures of total renal Klotho status.

#### iv) Is PT Klotho loss the main driver of aging-like phenotype and CKD?

Although Klotho expression per cell is modest in the PT and high in the DC, PT cells vastly outnumber DC cells in the kidney [5]. Therefore, the cumulative Klotho protein across the kidney is likely dominated by PT-derived Klotho. As discussed earlier, Klotho deletion that includes the PT invariably causes severe hyperphosphatemia, FGF23 resistance, hypervitaminosis-D and aging-like phenotype [1, 4, 5, 14]. In contrast, DC-specific Klotho deletion induces hypercalciuria and lower bone density without hyperphosphatemia or aging-like phenotype (Fig. 1c). These findings suggest that PT Klotho deficiency, through its effects on phosphate and vitamin D homeostasis, likely contributes to the aging-like phenotype. Convergent rescue experiments confirm this. Dietary Pi restriction, inactivation of NaPi-IIa, or inhibiting calcitriol rescued the phenotype in global Klotho knockout mice [12, 20]. Notably, *Fgf23* knockout mice develop an aging-like phenotype similar to PT-specific, kidney-specific, and global Klotho knockout mouse, underscoring a shared pathogenic mechanism of dysregulated Pi and vitamin D homeostasis [1, 4, 14, 21].

**Fig. 3:**
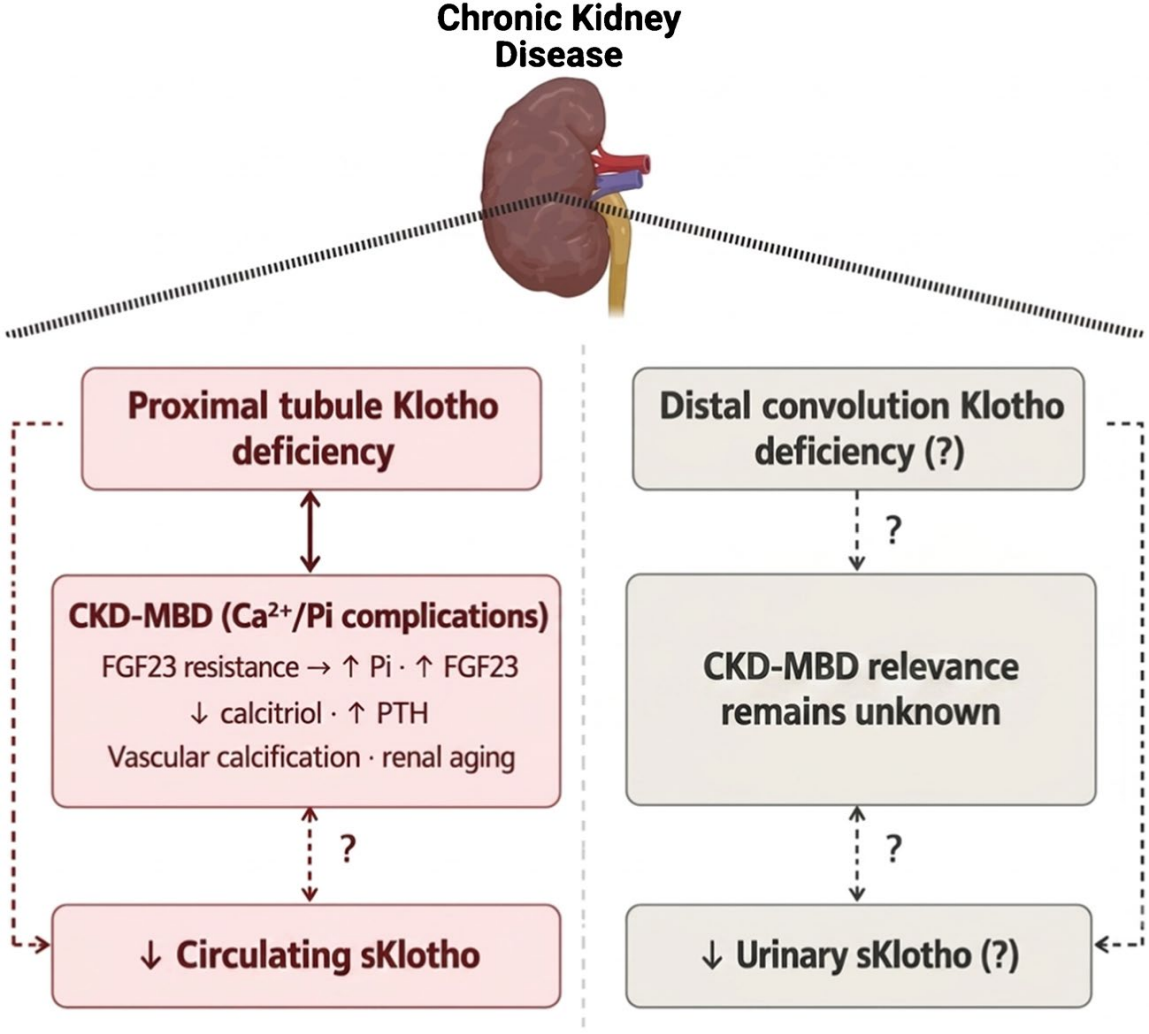
Proposed relationship between chronic kidney disease (CKD) and segment-specific Klotho deficiency. PT Klotho deficiency (left, red) is proposed as the primary driver of CKD-MBD. FGF23 resistance due to PT Klotho deficiency leads to hyperphosphatemia further elevating FGF23, which suppresses calcitriol and drives secondary hyperparathyroidism, probably contributing to vascular calcification and renal aging. The bidirectional solid arrow indicates that PT Klotho deficiency and CKD-MBD complications reinforce each other. By contrast, DC Klotho deficiency and its relevance to CKD-MBD remain unclear (right, grey). Dashed lines indicate proposed relationships.

CKD is associated with loss of renal Klotho, though whether this occurs in the PT, DC, or both remains unclear. The CKD phenotype characterized by hyperphosphatemia, vascular calcification, ectopic mineralisation, secondary hyperparathyroidism, maps onto PT-specific rather than DC-specific Klotho knockout phenotypes [5, 6, 14, 19] (Fig. 3). PT Klotho deficiency also likely underlies FGF23 resistance in CKD. As Klotho expression declines in the PT, tubular responsiveness to FGF23 becomes progressively blunted, such that hyperphosphatemia persists despite elevated circulating FGF23, leading to worsening of the CKD- mineral and bone disorder (CKD-MBD) phenotype (Fig. 3). The consequences of DC Klotho deficiency in CKD remain largely unresolved. At least one study in diabetic nephropathy links DC Klotho loss to hypercalciuria [22]. Segment-specific targeting of PT Klotho-dependent functions therefore represents a rational therapeutic approach in CKD. DC Klotho may become relevant in specific etiologies where hypercalciuria is prominent but is unlikely to address the core CKD-MBD phenotype.

## Conclusions

As Klotho’s systemic effects are primarily mediated through the kidneys, dissecting the segment-specific functions of Klotho is crucial for elucidating the mechanisms of mineral metabolism and CKD. Although Klotho protein was first discovered in the DC, it appears to exert its most consequential systemic effects through the PT. As an obligate co-receptor for FGF23-dependent Pi and vitamin D regulation, and likely the major source of circulating sKlotho, PT Klotho contributes to protection against aging-like and CKD phenotypes in mice. In contrast, DC Klotho represents a module that regulates the transcriptional program governing Ca^2^⁺ reabsorption and bone health while maintaining urinary sKlotho, whose function remains unclear. This dichotomy is likely non-coincidental: FGF23–Klotho signaling emerged in vertebrates with bony skeletons alongside the evolution of nephron segments capable of fine-tuning Pi and Ca^2^⁺ handling, placing the PT and DC at the centre of the bone-kidney axis. Clinically, PT Klotho loss likely contributes to hyperphosphatemia and may represent a key abnormality in CKD-MBD. Restoring PT Klotho function could therefore be an important therapeutic strategy for CKD-MBD.

## Data Availability

No datasets were generated or analysed during the current study.
